# Conditioned Medium of Human Amniotic Epithelial Cells Alleviates Experimental Allergic Conjunctivitis Mainly by IL-1ra and IL-10

**DOI:** 10.3389/fimmu.2021.774601

**Published:** 2021-11-22

**Authors:** Binxin Wu, Furong Gao, Jianhua Lin, Lixia Lu, Huiming Xu, Guo-Tong Xu

**Affiliations:** ^1^ Department of Ophthalmology of Shanghai Tenth People’s Hospital, Laboratory of Clinical Visual Science of Tongji Eye Institute, Tongji University School of Medicine, Shanghai, China; ^2^ Department of Obstetrics and Gynecology, Ren Ji Hospital, School of Medicine, Shanghai Jiao Tong University, Shanghai, China; ^3^ State Key Laboratory of Oncogenes and Related Genes, Renji-Med X Clinical Stem Cell Research Center, Ren Ji Hospital, School of Medicine, Shanghai Jiao Tong University, Shanghai, China; ^4^ Department of Pharmacology, Tongji University School of Medicine, Shanghai, China

**Keywords:** AECM, EAC, IL-1ra, IL-10, tissue stem cells

## Abstract

Allergic conjunctivitis (AC) is the most prevalent form of mucosal allergy, and the conditioned medium (CM) from mesenchymal stem cells has been reported to attenuate some allergic diseases. However, the therapeutic effects of CM from different tissue stem cells (TSC-CM) on allergic diseases have not been tested. Here, we studied the effects of topical administration of different human TSC-CM on experimental AC (EAC) mice. Only human amniotic epithelial cell-CM (AECM) significantly attenuated allergic eye symptoms and reduced the infiltration of immune cells and the levels of local inflammatory factors in the conjunctiva compared to EAC mice. In addition, AECM treatment decreased immunoglobulin E (IgE) release, histamine production, and the hyperpermeability of conjunctival vessels. Protein chip assays revealed that the levels of anti-inflammatory factors, interleukin-1 receptor antagonist (IL-1ra) and IL-10, were higher in AECM compared to other TSC-CM. Furthermore, the anti-allergic effects of AECM on EAC mice were abrogated when neutralized with IL-1ra or IL-10 antibody, and the similar phenomenon was for the activation and function of B cells and mast cells. Together, the present study demonstrated that AECM alleviates EAC symptoms by multiple anti-allergic mechanisms mainly *via* IL-1ra and IL-10. Such topical AECM therapy may represent a novel and feasible strategy for treating AC.

## Introduction

Allergic diseases have become a worldwide health problem and are increasing in prevalence. Among allergic diseases, allergic conjunctivitis (AC) is the most prevalent form of mucosal allergy and currently affects many people in the world (1), with symptoms of itching, redness of the mucosa, swelling of the eyelids, chemosis, and tearing (2). It is commonly believed that the allergen-driven T helper type 2 (Th2) immune response and type I hypersensitivity play crucial roles in the pathogenesis of AC (3). Current drugs for treating AC include antihistamines, mast cell (MC) stabilizers, or antihistamine/MC stabilizer combination, corticosteroids, and immunotherapy. However, long-term administration of antihistamine/MC stabilizers and corticosteroids may cause a variety of side effects, drug resistance, and intolerance (4, 5). For example, long-term use of olopatadine results in a 7% incidence of headache (6). Topical steroids can cause cataracts and glaucoma (5), as well as a risk of fungal infection and delayed healing of corneal epithelium (7). Although allergen immunotherapy is a safe and effective treatment for patients with AC (1), its effects on improving symptoms and clinical scores in allergic diseases are short term (8). Therefore, novel therapies for AC are required.

Tissue stem cells (TSC), including mesenchymal stem cells (MSC) and epithelial-shaped amniotic epithelial cells (AEC), possess the potential of self-renewal and multidirectional differentiation (9, 10), contribute to the regeneration of many tissues damaged by various diseases (11, 12), and exhibit immunomodulatory functions and inflammatory inhibitory effects in many immune-related diseases and inflammatory diseases in preclinical and clinical trials (11–14). In addition, previous studies have reported that MSC attenuate allergic diseases, such as asthma, allergic rhinitis, rheumatoid arthritis, and allergic skin disease (14, 15). Moreover, a recent study has shown that human AEC (hAEC) was effective in treating experimental allergic airway disease (16). Several molecular mechanisms have been reported for these phenomena: 1) immunomodulatory effects on immune cells, including inhibition of effector T cells, B cells, neutrophils, dendritic cells, and MCs; 2) promotion of the polarization of macrophages from type 1 to type 2 and upregulation of regulatory T (Treg) cell percentages (12, 17–19); and 3) the beneficial function of paracrine factors (20, 21).

Of note, TSC secrete many paracrine factors, including neurotrophic factors, growth factors, cytokines, receptors, chemokines, and anti-inflammatory factors to facilitate cell survival and regeneration and reduce inflammation response (20, 22). Mounting evidence has demonstrated that the immunomodulatory effects of TSC on immune cells are dependent on various soluble paracrine factors, including interleukin-1 receptor antagonist (IL-1ra), IL-10, transforming growth factor-β (TGF-β), indoleamine 2,3-dioxygenase (IDO), prostaglandin E2(PGE2) and C-C Motif chemokine Ligand 2 (CCL2) (15, 17, 23, 24). Therefore, application of conditioned medium (CM) from tissue stem cells (TSC-CM) on various diseases is reasonable. For allergic diseases, previous studies showed that the CM from MSC ameliorates allergic airway inflammation (25) and asthmatic pathological changes in asthmatic rats (26). Regarding AC, the CM from tumor necrosis factor-α (TNF-α)-pretreated mouse bone marrow MSC attenuates AC symptoms in the AC model (27). However, the pretreatment procedure with inflammatory factors may increase the complexity of the production process and bring safety concerns for clinical use. Therefore, exploring an appropriate CM without pretreatment process for the clinical treatment of AC and other allergic diseases is required.

On the other hand, preclinical and clinical trials have shown that TSC were effective in the treatment of ocular surface disorders by various transplantation methods, including intrastromal injection (28), subconjunctival injection (29), and cotransplantation with the amniotic membrane (30). However, from a clinical point of view, these cell transplantation methods are not appropriate for patients with AC. In contrast, topical administration is a common and acceptable method for patients with AC. Therefore, topical administration of CM was used in the study.

In the present study, we compared the therapeutic effects of topical administration of CM from human amniotic MSC (AMSCM), human bone marrow-derived MSC (BMSCM), human umbilical cord-derived MSC (UMSCM), human adipose-derived stem cells (ADSCM), and hAEC [amniotic epithelial cell-CM (AECM)] without pretreatment on AC model to find an efficient CM for the treatment of AC. Moreover, we investigated the underlying anti-allergic mechanisms of the effective CM and the vital paracrine factors in the CM for treating AC.

## Materials and Methods

### Isolation and Culture of TSC and Preparation of CM

hAEC, human amniotic MSC (hAMSC), human bone marrow-derived MSC (hBMSC), human umbilical cord-derived MSC (hUMSC), and human adipose-derived stem cells (hADSC) were isolated from different human tissues and cultured in the corresponding medium with 10% fetal bovine serum (FBS) and 1% penicillin/streptomycin (P/S) (all from Life Technology). The detailed protocol information is in the *supplementary materials and methods*. The collection and subsequent use of adult tissues were approved by the Human and Animal Research Ethics Committee of Renji Hospital, School of Medicine, Shanghai Jiaotong University. The people gave informed consent for sample collection.

For collecting the TSC-CM the cells were cultured in Dulbecco’s modified Eagle’s medium (DMEM)/F12 medium at passage 2 up to 90% confluency, washed with phosphate-buffered saline (PBS) three times, changed to basic medium DMEM/F12 and cultured for 24 h, and lastly harvested the CM and centrifuged at 2,000 rpm for 10 min at 4°C to remove cell debris. Next, we used a Pierce™ BCA protein assay kit (Thermo Fisher, USA) to measure total protein concentration of the CM and then normalized to the same concentration according to total protein concentration for the following experiments. Lastly, the normalized CM was aliquoted and stored at -80°C for use.

### Flow Cytometry

The stem cells were dissociated with trypsin, washed with cold PBS, and then separately stained with immunoglobulin G (IgG) or the following monoclonal antibodies conjugated to PE, PerCP, APC, or FITC: Epcam-PE, CD44-FITC, CD29-FITC, CD49f-FITC, CD73-FITC, CD105-APC, CD90-FITC, CD34-PerCP, CD31-FITC, CD45-FITC, and HLA-DR-FITC (all from eBioscience, USA). Upon being washed with PBS, the labeled cells were resuspended, and at least 10^5^ events were acquired by using a BD Accuri™ C6 flow cytometer (BD, USA).

### Adipogenic and Osteogenic Differentiation

The cells were cultured up to 80%–90% confluence and changed to the osteogenic differentiation medium or the adipogenic differentiation medium (STEMCELL Technologies, Vancouver, BC, Canada). Osteoblastic differentiation was analyzed by alizarin red staining. Adipocytic differentiation was identified by Oil Red-O staining.

### Topical Administration of the TSC-CM on Experimental Allergic Conjunctivitis (EAC) Mice 

Six-week-old BALB/c mice were purchased from Shanghai SLAC Co. Ltd. (Shanghai, China). All animal experiments were approved by the Committee on the Ethics of Animal Experiments of Tongji University (Permit Number: TJAA09620101). All procedures including animal eye studies were conducted in accordance with the “Statement for the use of animals in ophthalmic and vision research” of the Association for Research in Vision and Ophthalmology. The mice were randomly grouped into eight mice for each group. The groups were as follows: CON (normal control without administration of pollen and albumin, DMEM/F12 basic medium), EAC (experimental allergic conjunctivitis) (EAC, DMEM/F12 basic medium), DEX (EAC, TobraDex^®^ Dexamethasone Eye Drops from Alcon^®^), EAC+AECM, EAC+AMSCM, EAC+BMSCM, EAC+UMSCM, and EAC+ADSCM. The EAC model was induced according to previously reported methods with minor modification (31–33). In brief, BALB/c mice were immunized with 50 μg of short ragweed (SRW) pollen (Greer Lab, USA) and 5 μl albumin (Thermo Scientific, USA) by footpad injection on day 0 and day 5. The mice were then challenged with topical application of 10 μl SRW pollen solution (1.5 mg SRW pollen in PBS) in both eyes from days 10 to 14. Here, 10 μl CM was topically administered on both ocular surfaces of EAC mice four times daily from days 10 to 14 before challenge with SRW pollen solution (27). The clinical symptoms were given scores according to the standard previously described (31, 32), which includes conjunctival redness, eyelid edema, and mucus secretion under a slit lamp. The scratching response was defined as rapid movements of the paws toward the eye, as shown in **Supplementary Video 1**. The scratching times of each mouse 15 min after the challenge were also counted by observers who are blind to the treatment.

### Analysis of Soluble Factors in the TSC-CM

To measure the soluble factors in the TSC-CM, a protein antibody array was performed with a Raybiotech L-series human Antibody Array 507 (Raybiotech, Atlanta, GA, USA). The expression levels of 507 human target proteins including cytokines, chemokines, adipokines, growth factors, angiogenic factors, proteases, soluble receptors, soluble adhesion molecules, and other proteins in the TSC-CM were simultaneously detected (**Supplementary Tables 1–3**). CM from adult human foreskin fibroblasts (HEFCM) was used as control. The procedure was performed according to the manufacturer’s instructions. Finally, the glass slide was dried, and fluorescent signals were scanned with GenePix 4000B (Axon Instruments, GenePix version 5.0). For each array, the background was subtracted from the protein intensity values, and the values were scaled to the internal control and floored at 1 unit.

### Enzyme-Linked Immunosorbent Assay (ELISA)

The concentrations of IL-1ra and IL-10 in the AECM were measured by using human IL-1ra and IL-10 ELISA kit (R&D, USA). And the concentrations of IgE in ophthalmic lavage fluid or serum were tested by using mouse IgE ELISA kit (Elabscience, China). The histamine ELISA kit is from Elabscience. For ELISA kits, the assay range was 5–500 pg/ml.

### Testing the Function of IL-1ra and IL-10 in the EAC Mice

Based on the ELISA assay of AECM, we applied exogenous human recombinant IL-1ra and IL-10 with the concentration of 1,000 pg/ml and 200 pg/ml, respectively, on EAC mice. Moreover, we neutralized IL-1ra or IL-10 in AECM with IL-1ra and IL-10 antibodies, then administered on EAC mice. The groups were as follows: EAC, EAC+AECM, EAC+AECM+IL-1ra Ab, EAC+IL-1ra, EAC+AECM+IL-10 Ab, EAC+IL-10, and EAC+IL-1ra+IL-10. The EAC model and treatment were performed as described above.

### Histopathological Analysis

Fifteen days after sensitization, the mice were sacrificed, and the eyelid and conjunctival tissue were fixed and embedded in paraffin, then cut into sections with a thickness of 5 μm. For histopathological analysis, the sections were stained with hematoxylin and eosin (H&E), Giemsa, and toluidine blue, respectively. The sections were visualized with a microscope. For qualification analysis, at least six representative sections of the conjunctiva were counted. At least three mice were used in each group. Image J (Media Cybernetics, USA) was used for image analysis.

### Culture and Activation of B Cells

For collecting and isolating B cells, BALB/c mice were first immunized with 50 μg of SRW pollen and 5 μl albumin by footpad injection on day 0 and day 5, then splenocytes were isolated from mice at day 10, and red blood cells were lysed using RBC Lysis Buffer (Yeasen, China). Then, splenic B cells were isolated by B220^+^ antibody (BD Biosciences, USA) by positive selection according to the manufacturer’s instructions. Isolated B cells were cultured in RPMI 1640 (Sigma-Aldrich, USA) containing 10% FBS, 2 mM L-glutamine, 55 mM 2-mercaptoethanol, and 1% P/S. Then, B cells (1 × 10^6^/ml) were stimulated by the addition of IL-4 (50 ng/ml, Novoprotein, China) plus lipopolysaccharide (LPS; 10 mg/ml, Beyotime, China) to the culture medium for 5 days. The supernatants were collected for IgE analysis, and B cells were collected to measure cell viability.

### Culture and Activation of MC

The human MC line HMC-1 was obtained from the ATCC and were cultured in RPMI 1640 medium supplemented with 10% FBS and 1% P/S. For HMC-1 activation, the cells (1 × 10^6^/ml) were stimulated with phorbol 12-myristate 13-acetate (PMA, 20 nM, Selleck, USA) and calcium ionophore (A23187, 1 μM, Aladdin, China) (PMACI) (34) for 16 h. The cell supernatants were collected for ELISA analysis, and the cells were collected for real-time PCR and Western blot analysis.

### Intracellular Calcium Measurement

The intracellular calcium levels were measured according to the method described (35). In brief, (Ca^2+^i) the HMC-1 cells (5 × 10^6^ cells/ml) were preincubated with 1 mM of Fluo-8-AM (Keygen, China) for 30 min at 37°C. After washing twice with calcium-free medium (media with 3 mM EGTA), the cells were treated with CM for 24 h. Then, the cells were replated in a black 96-well plate to test Ca^2+^i. After stimulating with PMACI, the dynamic change of the Ca^2+^i recorded every 10 s using a fluorescent Microplate Reader (SpectraMax iD3, Molecular Devices, USA) at an excitation wavelength of 494 nm and an emission wavelength of 516 nm.

### Culture and Activation of Human Umbilical Vein Endothelial Cells

The HUVECs were cultured with endothelial cell medium (ECM, ScienCell, USA) supplemented with 10% FBS, 1% endothelial cell growth supplement, and 1% P/S. For HUVECs activation, the monolayer cells were stimulated by histamine (100 mM, Sigma-Aldrich, USA) for 30 min, then collected for Western blot analysis.

### Quantitative Real-Time PCR (RT-PCR)

The total RNA of cells was extracted using TRIzol reagent (Takara, Japan) and reverse transcribed into cDNA using the PrimeScript RT reagent kit (Takara, Japan). RT-PCR was performed with SYBR Green PCR Master Mix (Tiangen Biotech, China) and the following cycling parameters: denaturation at 95°C for 5 min followed by 40 cycles of 95°C for 30 s and 60°C for 30 s using BioRad CFX96TM RT-PCR detection system (BioRad, USA). It was normalized by the expression of GAPDH. The relative amount of each gene was measured using the 2^-ΔΔCT^ method. All quantitative RT-PCR experiments were performed at least three independent experiments. For mouse sample, the samples were harvested from at least three mice and the RT-PCR experiments were repeated three times. The information of the primers was listed in **Supplementary Table 4**.

### Western Blot Analysis

The cells were lysed using RIPA lysis buffer (Beyotime, China) containing protease and phosphatase inhibitor cocktails (Merck, USA). The protein concentration was determined by BCA protein assay kit. The PVDF membranes (Millipore, USA) were blocked with 5% nonfat milk or 5% bovine serum albumin (BSA) and incubated with primary antibodies overnight at 4°C. After washing with TBST, the membranes were incubated with corresponding HRP-conjugated secondary antibodies (Proteintech, China) for 1 h at room temperature. Densitometric analysis of proteins was performed by Tanon 5200S (Tanon, China). GAPDH antibody was used as an internal control, while Lamin B was used as internal control of nuclear protein. Primary antibodies used are listed in **Supplementary Table 5**.

### *In Vivo* and *In Vitro* Vascular Permeability Assay

*In vivo* vascular permeability assays were performed according to previously reported methods (36) with minor modification. First, the mice were injected through the tail vein with 0.5% Evans blue dye solution in PBS (12 ml/kg); the mice were then photographed 1 h after injection. Next, the eyelid and conjunctival tissue were incubated in formamide at 55°C for 2 days to extract Evans blue dye. The extract was centrifuged twice at 10,000 ×g for 20 min at 4°C. The concentration of Evans blue dye in the extract was tested at 620 nm to evaluate the vascular permeability.

*In vitro* vascular permeability assays were performed as previously described (37). In brief, HUVECs were grown in 24-well Transwell filters (Millipore, USA) in 500 μl medium to form a cell monolayer. After adding 7.5 μl of streptavidin-HRP (1.5 mg/ml, Beyotime, China) to the upper chamber for 8 h, the monolayer permeability of the cells was tested by stimulation with 100 mM histamine for 30 min. Finally, streptavidin-HRP was added to the upper cell monolayer for 5 min, and the lower cell supernatant (500 μl) was collected. HRP activity was detected with TMB substrate. HRP activity was measured at OD 450 nm to evaluate the permeability of the cells.

### Determination of Transendothelial Electrical Resistance (TEER)

The TEER of HUVECs was determined by Millicell-ERS2 Volt-Ohm Meter (Millipore, USA) according to the manufacturer’s protocol. Briefly, HUVECs (2 × 10^4^ cells per well) were plated onto 24-well Transwell filters to form a cell monolayer. The TEER values of the monolayers were measured with electrodes. TEER values (Ω·cm^2^) were calculated by subtracting the resistance of cell-free filter and then corrected according to the culture surface area.

### Statistical Analysis

The data are presented as mean ± SEM of at least three independent experiments, statistical analysis was assessed by SPSS software 22.0, and statistical significance was determined using Student’s *t*-test for comparison of three groups and one-way ANOVA with Tukey’s multiple comparison test for multiple comparisons. Statistical significance was set as ^*^ *P* < 0.05, ^**^ *P* < 0.01, and ^***^ *P* < 0.001, unless otherwise indicated.

## Results

### Comparison of the Effects of the TSC-CM on EAC

First, human different types of TSC were isolated and cultured according to the protocol in the *Materials and Methods* section. The tested TSC were identified by flow cytometry (**Supplementary Figure 1**), and the differentiation ability of the TSC was evaluated by their adipogenic and osteogenic potentials. **Supplementary Figure 2** shows that the adipogenic and osteogenic potentials were comparable, except for hADSC and hBMSC. hADSC produced more adipose globelets, and hBMSC formed more bone-like nodules, revealing that the differentiation potentials of TSC are related to tissue origin of stem cells. These results demonstrated that the TSC had high purity and good viability, indicating that CM could be collected from the TSC for the subsequent experiments.

**Figure 1:**
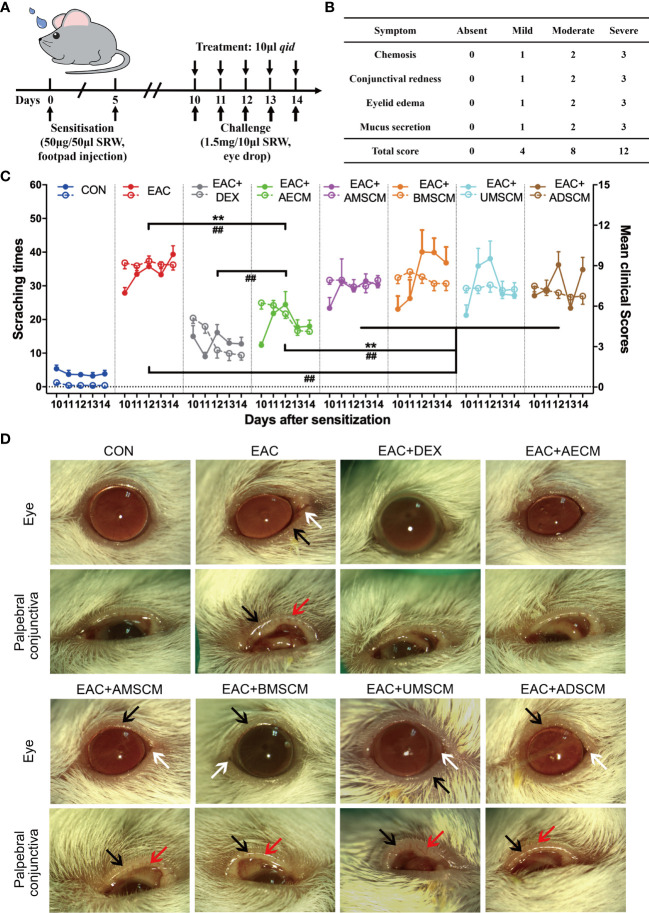
Comparison of the therapeutic effects of the TSC-CM on EAC mice. **(A)** A schematic illustration of the experimental design. Mice were first immunized with 5 μl SRW solution at day 0 and day 5, then challenged with 10 μl SRW solution daily from day 10 to day 14. At the same time, the mice were pretreated with 10 μl TSC-CM four times per day. **(B)** Clinical score was used to evaluate the severity of chemosis, conjunctival redness, eyelid edema, and mucus secretion under slit lamp. **(C)** The times of scratching response (left Y axis, solid points) and clinical scores (right Y axis, hollow points) of the severity of EAC symptoms were evaluated at the indicated time points after challenge with SRW solution in different groups. CON represents the normal control mice pretreated with DMEM/F12 medium without SRW pollen treatment. Solid points represent the times of scratching response. Hollow points represent clinical scores. The data are expressed as mean ± SEM, n = 8. ^**^ *P* < 0.01 in scratching times. ^##^ *P* < 0.01 in clinical scores. **(D)** Representative images of ocular symptoms in each group photographed by slit lamp microscopy 24 h after the last challenge. White arrows represent chemosis, red arrows represent conjunctival redness, and black arrows represent eyelid edema.

EAC was induced by SRW pollen as previously described (31–33). Mice were then challenged with SRW solution daily from day 10 to day 14 when different treatments were given. In the CM-treated groups, TSC-CM was applied to the ocular surface of EAC mice before the daily challenge (**Figure 1A**). The CM remained on the ocular surface for approximately 15 min. The severity of EAC symptoms was assessed by the chemosis, conjunctival redness, eyelid edema, and mucus secretion scores (**Figure 1B**) as well as scratching response times according to previous reports (31–33). All clinical scores and scratching response times were significantly increased in the EAC group compared to the control group without SRW induction (**Figure 1C**). AECM treatment significantly reduced the times of scratching responses and the severity of clinical scores compared to those of the EAC group with basic medium instead of CM (*P* < 0.01). Moreover, the reduction of scratching response times was comparable in AECM group to that of the DEX group (used as a positive control); however, the reduction of clinical scores in AECM group was slightly worse than that of DEX group, indicating that the therapeutic effect of AECM is slightly weaker than that of DEX. In addition, although AMSCM, BMSCM, UMSCM, and ADSCM slightly reduced clinical scores compared to the EAC group, but not scratching responses, their effects were weaker than those of AECM group.

To record and analyze the symptoms of EAC mice, mouse eyes were photographed on day 15. As shown in **Figure 1D**, mice in EAC group displayed severe signs of AC, such as evident hyperemia and edema in the conjunctiva compared to the control group. Consistent with the above observations, AECM significantly reduced conjunctival redness and eyelid edema of mice in AECM-treatment EAC mice compared to EAC mice, while AMSCM and BMSCM only slightly alleviated AC symptoms of the mice. The above results indicated that AECM significantly ameliorates AC induced by SRW pollen. Thus, we focused on AECM in subsequent experiments.

### AECM Reduces Inflammatory Response in EAC Mice

**Figure 2:**
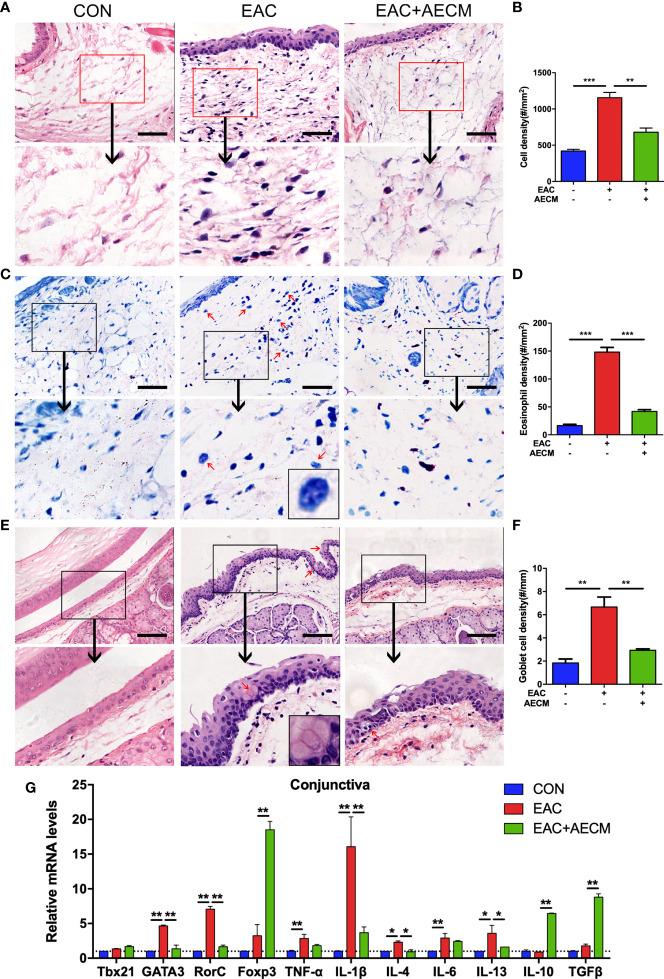
AECM treatment alleviated inflammatory response in EAC mice. **(A)** Representative image of H&E staining of the conjunctiva in mice in different treatment groups. Scale bar, 50 μm. **(B)** Quantification of cell density in the conjunctiva of mice in each group. The data are expressed as the mean ± SEM. At least six representative sections of the conjunctiva were counted. At least three mice were used in each group. **(C)** Giemsa staining conjunctiva of mice in different treatment groups. Red arrows indicate eosinophils. Scale bar, 50 μm. The magnification black box at the bottom of the middle pattern represents a large eosinophil with bilobed nucleus. **(D)** Quantification of eosinophil density of the conjunctiva of mice in each group. **(E)** H&E staining of conjunctiva of mice in different treatment groups. Red arrows indicate goblet cells with vacuole. Scale bar, 100 μm. The magnification black box of the middle pattern represents a magnification image of goblet cells with vacuole. **(F)** Quantification of goblet cell density in the conjunctiva of mice in each group. **(G)** Real-time PCR analysis of mRNA levels of cytokines and marker genes of immune cells in conjunctiva of mice in different groups. The mRNA levels of genes in CON group were set as 1, and the data are expressed as the mean ± SEM, n = 3. *P < 0.05, **P < 0.01, and ***P < 0.001.

To investigate the involvement of inflammation in the EAC model and the effects of AECM in its intervention, we performed histopathological examination of the conjunctiva of EAC mice. As shown in **Figures 2A–D**, the infiltration of eosinophils and other immune cells in the conjunctiva increased in EAC mice but significantly decreased in the AECM-treatment EAC mice compared to basic medium-treatment EAC mice. When treated with AECM, the immune cells and eosinophils in the conjunctival tissue of mice were reduced by 28.23% ± 5.1% and 60% ± 4.5%, respectively, compared to EAC mice (**Figure 2D**). When conjunctival goblet cells (one typical type of mucus-secreting cells) (38–40) were examined, similar patterns of the SRW-induced increase in EAC group and the inhibitory effects of AECM were observed (**Figures 2E, F**).

To determine whether AECM treatment affects the local inflammatory environment in the conjunctiva of EAC mice, we examined the conjunctiva from different groups by real-time PCR. Compared to the control group, the SRW challenge increased the expression of marker genes associated with inflammatory subsets of CD4^+^ T cells, such as Th2 (GATA3) and Th17 (RorC), and the corresponding inflammatory cytokines, including TNF-α, IL-1β, IL-4, IL-6, and IL-13, in the conjunctiva of mice, but AECM treatment significantly reduced the expressions of these marker genes and cytokines (**Figure 2G**). In contrast, AECM treatment significantly increased the levels of the anti-inflammatory factors IL-10 and TGF-β, as well as the expression of Foxp3 (**Figure 2G**). In addition, we also detected the expression of the genes related to the inflammatory milieu in cervical lymph nodes of mice and obtained similar results to those of the conjunctiva (**Supplementary Figure 3**). The above results demonstrated that AECM significantly reduces the allergic inflammatory response by reducing the infiltration of inflammatory immune cells, decreasing the levels of inflammatory factors and increasing the levels of anti-inflammatory factors.

### IL-1ra and IL-10 Anti-Inflammatory Factors Are Abundant in AECM

**Figure 3:**
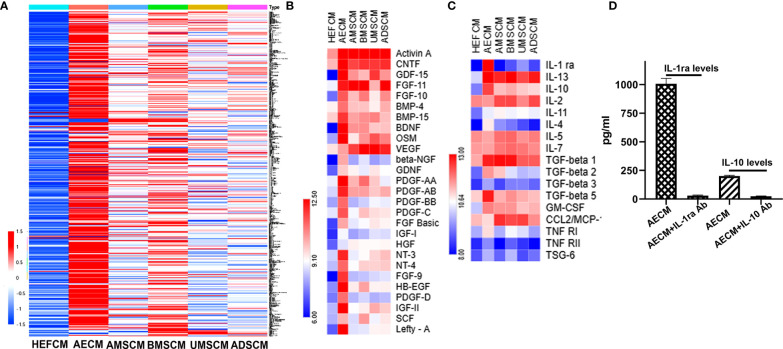
Analysis of paracrine factors secreted by TSC. **(A)** Soluble factor in the AECM, MSC, AMSCM, BMSCM, UMSCM, and ADSCM were analyzed by a human antibody array 507. HEFCM was used as a control. The normalized array data of 507 proteins in the CM of different tissue stem cells were analyzed by SAM, and the relative concentrations of these factors were shown as a “heat map”. **(B)** The relative concentrations of growth factors were shown as a “heat map”. **(C)** The relative concentrations of anti-allergic-related factors including anti-inflammatory factors and TGF-β were shown as a heat map. **(D)** ELISA analysis of the concentration of IL-1ra and IL-10 in the AECM and their levels when neutralized with their antibodies.

To understand the molecular mechanism of AECM in ameliorating EAC, we performed a protein chip assay of 507 protein factors (**Supplementary Table 1**) to analyze the CM tested in this study, and the HEFCM was used as a control. The relative expression levels of the paracrine factors in the CM are shown as a heat map in **Figure 3A**. The paracrine factors in AECM were generally higher than those in other TSC-CM and the control HEFCM. In addition, we also noticed that many growth factors and cell adhesive molecules in AECM have similar phenomena (**Figure 3B**). Focusing on the anti-inflammatory factors, TGF-β and CCL2, which is related to the anti-allergic function of the stem cells (15), we observed that the relative levels of IL-1ra and IL-10 were higher in AECM than those in the other TSC-CM with concentrations of 1,007 ± 105 pg/ml and 200 ± 15.8 pg/ml, respectively (**Figures 3C, D**). Thus, we applied exogenous human recombinant IL-1ra and IL-10 with the same concentrations 1,000 pg/ml and 200 pg/ml, respectively, in the subsequent experiments. Moreover, we confirmed that IL-1ra and IL-10 antibodies did neutralize IL-1ra and IL-10 in AECM (**Figure 3D**). These data revealed that IL-1ra and IL-10 are abundant in AECM, and they may be important paracrine factors in AECM that mediate its inhibitory effects on EAC.

### AECM Inhibits Immunoglobulin E Release by B Cells

**Figure 4:**
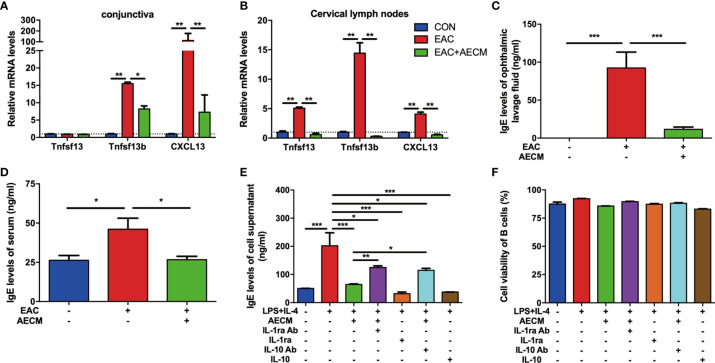
AECM inhibited IgE release by B cells. **(A, B)** Real-time PCR analysis of mRNA levels of marker genes of B cells in conjunctiva **(A)** and cervical lymph nodes **(B)** of mice in each group. The expression levels of genes in CON group were set as 1, and the data are expressed as the mean ± SEM, n = 3. **(C, D)** IgE levels in the ophthalmic lavage fluid **(C)** and serum **(D)** of mice in each group. The data are expressed as the mean ± SEM, n = 3. **(E)** IgE levels in the supernatant of purified B cells activated by IL-4 (50 ng/ml) plus LPS (10 mg/ml) for 5 days in each group. Data were collected from at least three separate experiments, and the data are expressed as the mean ± SEM. **(F)** The cell viability of purified B cells was assayed by trypan blue exclusion. *P < 0.05, **P < 0.01, and ***P < 0.001.

Considering the possible role of B cell activation and IgE production in allergic diseases (41, 42), we examined the recruitment and activation of B cells and IgE production in EAC mice with or without AECM treatment. As shown in **Figure 4A**, both CXCL13 (a potent chemokine for B cells) (43) and Tnfsf13b (a potent B cell-activating factor) were highly expressed in the EAC group, indicating that B cells could be activated and then release effective chemokines. However, AECM treatment decreased the expression of CXCL13 and Tnfsf13b. Additionally, AECM also inhibited the expression of CXCL13, Tnfsf13b, and Tnfsf13 (a potent B cell-proliferative factor) in cervical lymph nodes (**Figure 4B**), suggesting that AECM treatment inhibits B-cell proliferation and maturation in cervical lymph nodes, leading to B-cell migration into the conjunctiva of EAC mice. When the ophthalmic lavage fluid and the serum of mice in each group were collected and analyzed by ELISA, the IgE concentration in the ophthalmic lavage fluid and the serum of EAC mice increased but were both significantly reduced by AECM treatment (**Figures 4C, D**). In an *in vitro* experimental system, the level of IgE released from isolated B cells derived from BALB/c mice immunized with SRW was increased after stimulation with LPS+IL-4 but was maintained at a low level when cells were pretreated with AECM (**Figure 4E**), while B-cell viability remained unaffected (**Figure 4F**).

As a continuous effort to explore the roles of IL-1ra and IL-10 in the intervention of AECM in EAC mice, the effects of IL-1ra and IL-10 on B-cell activation and function were examined. As shown in **Figure 4E**, the addition of either recombinant IL-1ra or IL-10 at the concentration of 1,000 pg/ml and 200 pg/ml, respectively, mimicked the inhibitory effect of AECM on IgE production, but adding anti-IL-1ra or IL-10 antibodies to AECM partially neutralized the inhibitory effects of AECM on IgE production. It is worth noting that the B-cell viability was not affected by any of the treatments (**Figure 4F**). These results suggested that IL-1ra and IL-10 may be the key factors in AECM due to their inhibitory effects on the activation and function of B cells.

### AECM Inhibits MC Activation and Function

**Figure 5:**
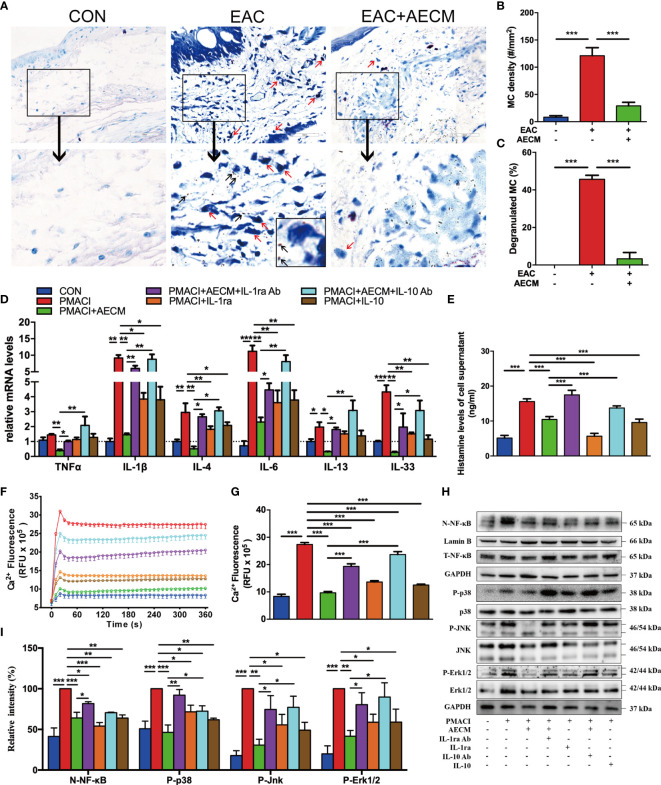
AECM inhibited MC function. **(A)** Representative images of toluidine blue staining of MC in the conjunctiva of mice in different groups. The red arrows represent the dark blue staining of the degranulated MC, surrounded by many small dark particles (black arrow). Scale bar, 50 μm. **(B, C)** Quantification of the MC **(B)** and degranulated MC **(C)** in the conjunctiva of mice in each group. At least six representative sections of the conjunctiva were counted. At least three mice were used in each group. **(D)** The mRNA levels of cytokines in HMC-1 cells in each group as indicated. Data were collected from at least three separate experiments, and the data are expressed as the mean ± SEM. **(E)** Histamine concentration in the supernatant of HMC-1 cells in different groups as indicated by ELISA. **(F)** Ca^2+^i response in HMC-1 cells in different groups. **(G)** Qualification of dynamic changes of Ca^2+^i in HMC-1 cells in different groups over 180 s. **(H)** Western blot analysis of the protein levels of nuclear and total NF-κB, p-P38 and P38, p-JNK and JNK, p-Erk1/2 and Erk1/2 in different treated HMC-1 cells. The level of Lamin B was used as an internal control of nuclear protein, and GAPDH was used as an internal control of the total protein of the cell lysate. N-NF-κB represents nuclear NF-κB. **(I)** Qualification of relative protein levels of N-NF-κB, p-P38, p-JNK, and p-Erk1/2. *P < 0.05, **P < 0.01, and ***P < 0.001.

We next investigated the effect of AECM on MC activation and functions. Toluidine blue staining showed that the numbers of MC and percentages of degranulated MC in the conjunctiva were increased in EAC mice compared to those in the control group and reduced by AECM treatment compared to those in EAC group (**Figures 5A–C**). These data indicated that AECM treatment significantly reduces the enrichment and activation of MC in the conjunctiva of EAC mice. Furthermore, we investigated the mechanism of AECM in an *in vitro* system using HMC-1 cells, an immortalized MC line. After 24 h of pretreatment with AECM, HMC-1 cells were stimulated with PMACI (34) for an additional 16 h. As shown in **Figure 5D**, the mRNA levels of TNF-α, IL-1β, IL-4, IL-6, IL-13, and IL-33 inflammatory factors were increased under PMACI stimulation, and AECM treatment significantly decreased the levels of these inflammatory factors. The addition of recombinant IL-1ra and IL-10 showed AECM-like inhibitory effects, and the addition of their neutralizing antibodies to AECM significantly abrogated the inhibitory effects of AECM. Similar to the patterns of AECM effects on the expression of inflammatory factors, histamine release by activated HMC-1 cells increased three-fold under PMACI stimulation but was reduced by AECM treatment (**Figure 5E**). When either IL-1ra or IL-10 was added to the PMACI-treated cells, a similar reduction in histamine release of AECM-treatment cells was observed. Furthermore, this effect of AECM was significantly offset when treated with AECM neutralized by IL-1ra or IL-10 antibodies (**Figure 5E**). Similar to the B-cell results, the MC viability was not affected (**Supplementary Figure 4**). These data suggested that AECM also inhibits the activation and function of MC.

We next investigated the molecular mechanisms of AECM-mediating MC inhibition. Previous studies have demonstrated that IgE binds to the surface receptor (FcεRI) of MC and induces calcium mobilization, leading to MC degranulation and histamine release (35, 39, 44). Consistently, exposure to PMACI resulted in increased Ca^2+^i in HMC-1 cells, while AECM pretreatment maintained the Ca^2+^i concentration at the control level (9.68 ± 0.19 *vs*. 27.37 ± 0.29 RFU × 10^5^, *P* < 0.01; **Figures 5F, G**). Nevertheless, the inhibitory effects of AECM were weakened by adding anti-IL-1ra or IL-10 antibodies to AECM. In addition, human recombinant IL-1ra and IL-10 also showed an inhibitory effect on Ca^2+^ mobilization, although weaker than that of AECM, suggesting that IL-1ra and IL-10 also participate in the suppressive effect of AECM on MC calcium mobilization.

Despite previous reports on the relationship between Ca^2+^i/ NF-κB signaling and inflammatory/allergic responses (45–47), we hypothesized whether the regulatory effect of AECM on MC is mediated by NF-κB signaling. Thus, we performed Western blotting to examine NF-κB signaling and related signaling molecules. As shown in **Figures 5H, I**, the level of NF-κB was increased in MC exposed to PMACI but decreased when treated with AECM. Adding IL-1ra or IL-10 antibody to AECM impaired the effects of AECM on NF-κB signaling, while only recombinant IL-1ra or IL-10 could not completely counteract the effects of PMACI. In addition, the MAPK signaling pathway has been reported to be related to inflammatory/allergic responses (48). Similar to the effects of AECM on NF-κB signaling, AECM treatment downregulated the expression of p-P38, p-JNK, and p-Erk activated by PMACI. However, such downregulation was abrogated in the presence of IL-ra or IL-10 antibody in AECM. Furthermore, recombinant IL-1ra or IL-10 could reduce the expression of p-P38, p-JNK, and p-Erk expression compared to that in the PMACI group. Taken together, these finding suggested that AECM inhibits MC function by regulating Ca^2+^i/NF-κB and MAPK signaling pathways.

### Antihistamine Effects and Mechanism of AECM in Ameliorating EAC

**Figure 6:**
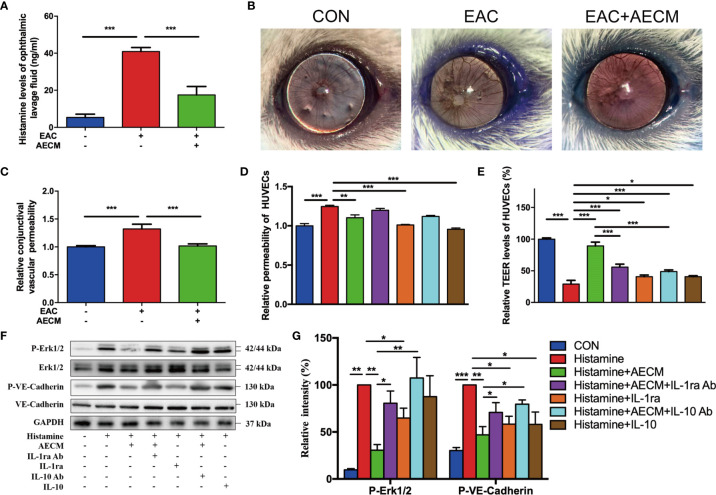
Antihistamine effects of AECM. **(A)** Histamine levels in ophthalmic lavage fluid of mice in different groups measured by ELISA. **(B)** Conjunctival vascular permeability of EAC mice was tested by Evans blue. Representative images were represented. **(C)** Quantification of conjunctival vascular permeability of EAC mice in different groups. n = 3. **(D)** Relative permeability of HUVECs in different treatment groups as indicated. **(E)** The TEER level of HUVECs in different groups. **(F)** Western blot analysis of protein level of p-Erk1/2 and Erk1/2, p-VE-cadherin and VE-cadherin in different treated HUVECs. The level of GAPDH was used as an internal control of the total protein of the cell lysate. **(G)** Qualification of relative protein levels of p-Erk and p-VE-cadherin. *P < 0.05, **P < 0.01, and ***P < 0.001.

To further understand the potential molecular mechanism of AECM, we investigated the possible effects of AECM on histamine. As expected, SRW activated histamine production in the ophthalmic lavage fluid of EAC mice (**Figure 6A**). Furthermore, AECM treatment decreased the histamine levels in the ophthalmic lavage fluid samples of mice. Conjunctival vascular permeability was also examined using Evans blue dye (36). The results showed that the conjunctival vascular permeability was significantly increased in EAC mice, but AECM treatment maintained permeability at the same low level as that of the control mice (**Figures 6B, C**). We also used HUVECs to evaluate permeability according to a previous method (37). Exposure to histamine increased the permeability of HUVECs, and this increase was prevented by AECM treatment (**Figure 6D**). In addition, recombinant IL-1ra and IL-10 significantly reduced the permeability of HUVECs stimulated by histamine, while the addition of IL-1ra or IL-10 antibody to AECM counteracted the effect of AECM (**Figure 6D**). To confirm this observation, TEER was also used to evaluate vascular permeability and integrity. As shown in **Figure 6E**, exposure to histamine decreased the TEER levels in HUVECs, while AECM restored the TEER levels. However, recombinant IL-1ra or IL-10 only showed a mild effect in restoring the TEER levels, and the effects of AECM with the IL-1ra or IL-10 antibodies were also mild.

To understand the molecular mechanism of AECM in maintaining vascular permeability and integrity, we examined signaling molecules potentially related to AECM, such as VE-cadherin and Erk, in HUVECs under different treatments (49). As shown in **Figures 6F, G**, histamine significantly increased phosphorylated VE-cadherin of HUVECs, which was reduced by AECM. Similarly, phosphorylated Erk was also increased in the cells under histamine treatment but was significantly decreased by AECM. Furthermore, the phosphorylation levels of VE-cadherin and Erk increased after AECM treatment with IL-1ra or IL-10 antibody and decreased after treatment with recombinant IL-1ra and IL-10. Overall, these results demonstrated that AECM inhibited histamine production and reduces vascular permeability by inhibiting the Erk/VE-cadherin pathway partially mediated by IL-ra and IL-10.

### IL-1ra and IL-10 Are Necessary for AECM to Alleviate EAC

**Figure 7:**
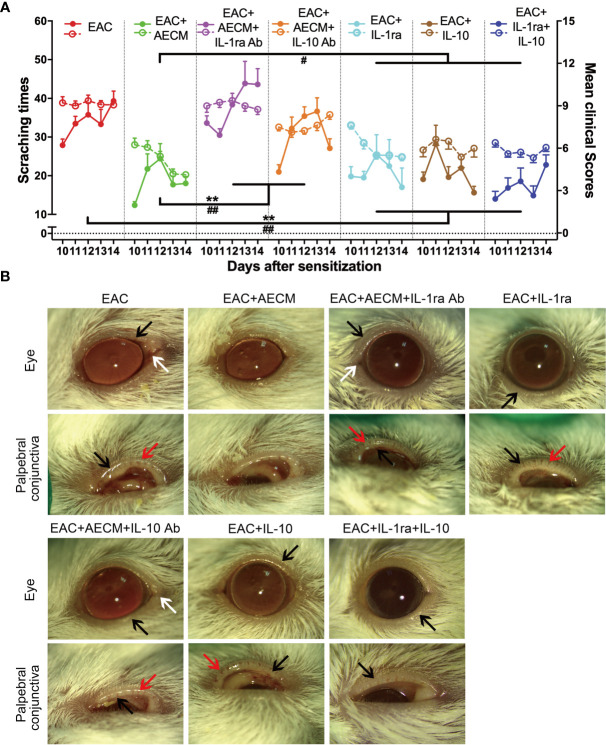
Anti-inflammatory factors IL-1ra and IL-10 are necessary for AECM-mediated amelioration on AC symptoms of EAC mice. **(A)** The times of scratching response (left Y axis, solid points) and clinical scores (right Y axis, hollow points) of the severity of AC symptoms of EAC mice in different groups were evaluated at the indicated time points after challenge with SRW solution. Solid points represent the times of scratching response. Hollow points represent clinical scores. The results are expressed as mean ± SEM. n = 8. ^**^ *P* < 0.01 in scratching times. ^#^ *P* < 0.05 and ^##^ *P* < 0.01 in clinical scores. **(B)** Representative images of ocular symptoms evaluated by slit lamp microscopy for each group at 24 h after the last challenge. White arrows represent chemosis, red arrows represent conjunctival redness, and black arrows represent eyelid edema.

Based on the above findings that IL-1ra and IL-10 mediate the inhibitory effects of AECM on B-cell and MC functions, we next examined whether IL-1ra and IL-10 are involved in the effects of AECM on EAC. As shown in **Figure 7**, the beneficial effects of AECM on EAC mice were completely reversed when mice were treated with AECM containing an IL-1ra or IL-10 neutralizing antibody, as evidenced by significantly increased scratching response times, clinical scores, conjunctival redness, and eyelid edema of mice (**Figures 7A, B**). Topical administration of either IL-1ra or IL-10 or IL-1ra+IL-10 partly mimicked the effects of AECM because the reduction in scratching response times was comparable to that of AECM group, but the reduction in clinical scores, conjunctival redness, and eyelid edema was worse than those of AECM (**Figures 7A, B**).

**Figure 8:**
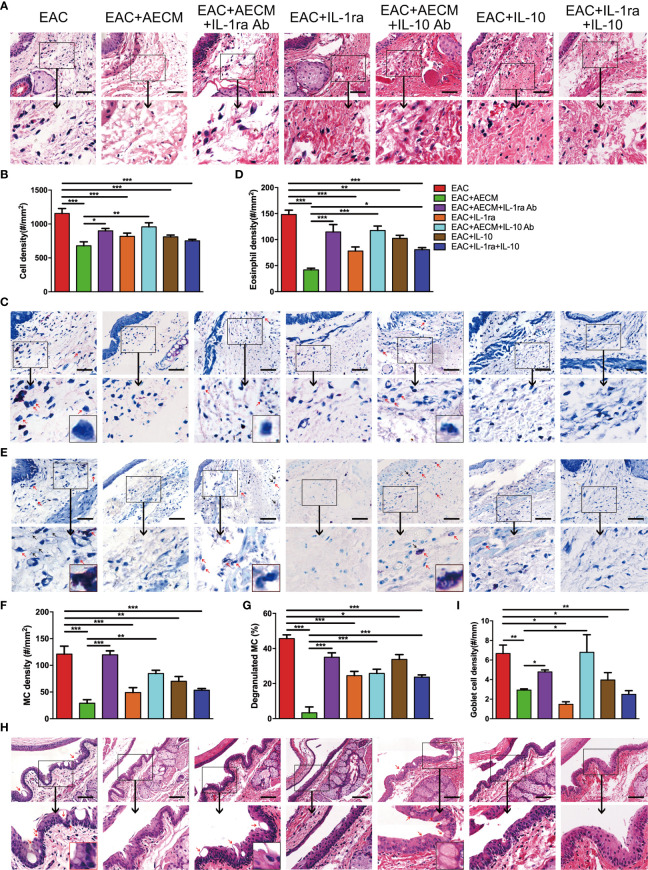
Anti-inflammatory factor IL-1ra and IL-10 are necessary for AECM-mediated reduction on the inflammation response in EAC mice. **(A)** Representative image of H&E staining of the conjunctiva of mice in different treatment groups. Scale bar, 50 μm. **(B)** Quantification of cell density in the conjunctiva of mice in different groups. The data are expressed as the mean ± SEM. At least six representative sections of the conjunctiva were counted. At least three mice were used in each group. **(C)** Giemsa staining of conjunctiva of mice in different treatment groups. Red arrows indicate eosinophils. Scale bar, 50 μm. **(D)** Quantification of eosinophil density in the conjunctiva of mice in different groups. **(E)** Representative images of toluidine blue staining of MCs in the conjunctiva of mice in different groups. Scale bar, 50 μm. **(F, G)** Quantification of the MC **(F)** and degranulated MC **(G)** in the conjunctiva of mice in different groups. **(H)** H&E staining of the conjunctiva of mice in different treatment groups. Red arrows indicate goblet cells with vacuole. Scale bar, 100 μm. **(I)** Quantification of goblet cell density in the conjunctiva of mice. *P < 0.05, **P < 0.01, and ***P < 0.001.

Finally, we examined the inflammatory profiles and the activation of MC in the conjunctiva of the mice under different treatments. As shown in **Figure 8**, inflammatory cell infiltration (**Figures 8A, B**), eosinophil accumulation (**Figures 8C, D**), MC enrichment and activation (**Figures 8E–G**), and goblet enrichment (**Figures 8H, I**) in the conjunctiva of EAC mice were all increased. Such increases were all reduced by AECM treatment. Administration of IL-1ra or IL-10 or IL-1ra+IL-10 showed less inhibitory effects than AECM, and the effects of AECM were reversed by treatment with AECM containing IL-1ra or IL-10 antibodies. Of note, the inhibitory effects of combination of IL-1ra and IL-10 were like that of IL-1ra or IL-10 on EAC and inflammatory cells, indicating there is no synergic effect between IL-1ra and IL-10 (**Figures 7**, **8**). These results indicated that the beneficial effects of AECM on EAC may be mainly due to its higher levels of IL-1ra and IL-10.

## Discussion

Researchers have found that MSC and AEC have anti-allergic effects on allergic diseases (15, 16), and the paracrine factors secreted by these TSC play important roles in immunoregulation or anti-inflammation (12, 22, 24–26, 50). Previous studies showed that MSC-CM is effective on some allergic diseases (25, 26). As for AC, topical administration of CM from TNF-α-stimulated mouse bone marrow MSC attenuates EAC (27). However, TNF-α-stimulated MSC increases the complexity of CM collection process and cost, including the addition of TNF-α and removal of TNF-α using a TNF-α antibody. In the present study, we evaluated the AECM and several types of MSC for their effects on EAC mice and found that only AECM significantly ameliorated AC symptoms than EAC group and AMSCM, BMSCM, UMSCM, and ADSCM. However, all the tested MSC-CM only slightly alleviated AC symptom of EAC mice, and their effects are evidently weaker than those of the AECM. As for the anti-allergic mechanism, AECM reduced the infiltration of inflammatory cells, including Th2 cells, B cells, eosinophils, and MC, and it suppressed the release of inflammatory factors. Of note, AECM treatment increased the levels of the anti-inflammatory factors IL-10 and TGF-β, as well as the expression of Foxp3. In addition, AECM also reduced the IgE production, histamine release, and vascular hyperpermeability. Because IgE plays important roles in the early phase of allergic diseases and vascular hyperpermeability, which is involved in the late inflammatory response (3, 41, 42, 51), the effects of AECM in this study suggested that AECM is effective in both the early phase and the late stage. In addition, AECM also inhibited goblet cell mucus secretion in EAC mice. Therefore, these finding suggested that AECM may be a better option for the treatment of AC or other allergic diseases.

Intriguingly, among all TSC tested, only AECM effectively attenuated allergic eye symptoms, which may be related to the function of the amniotic membrane. The amniotic membrane protects the developing embryo against various stimuli from the surroundings and suppresses the semiallogeneic immune response of the mother’s immune system (52). Due to its anti-inflammatory and immunosuppressive properties, human amniotic membrane tissue has long been used in the clinical treatment of burns and skin ulcers (52, 53). Moreover, hAEC is derived from the epithelial layer of the amniotic membrane, which is the layer closest to the embryo, and thus may have a strong immunosuppressive ability.

As for the molecular mechanism of AECM, we found that the relative levels of IL-1ra and IL-10 are higher in AECM than in the CM from other TSC and are the most abundant anti-inflammatory factors in the AECM, which may play crucial roles in mediating the anti-allergic effects of AECM on EAC. This conclusion was strongly supported by both *in vivo* and *in vitro* evidence in this study. Recombinant IL-1ra and IL-10 partially mimicked the effects of AECM on EAC, B cell, and MC function. In contrast, antibodies against IL-1ra or IL-10 significantly reduced the therapeutic effect of AECM on EAC mice and the inhibitory effects of AECM on B-cell IgE secretion and MC histamine release. Of note, IL-1ra and IL-10 could partially inhibit elevated Ca^2+^i stimulated by PMACI and Ca^2+^-induced responses in MC. As for the possible molecular mechanism, previous studies have shown that IL-1 binds to its receptor and activates numerous signaling pathways, including PLC, PKC, NF-κB, and MAPK pathway (54–56). While PLC and PKC can promote Ca^2+^ mobilization, leading to increased Ca^2+^i (57). Thus, IL-1ra competitively binds IL-1 receptor, thereby reducing IL-1-induced Ca^2+^ mobilization and activation of downstream NF-κB and MAPK pathway. Regarding IL-10, it is found to suppress the expression of FcϵRI, the allergen-specific IgE receptor on MC, thus inhibiting the activation of FcϵRI-binding tyrosine kinase Lyn and Syk and inhibiting Ca^2+^ mobilization (58). In addition, IL-10 can inhibit the generation of IL-1β (59), accordingly inhibiting IL-1-induced Ca^2+^ mobilization. However, more research is required to figure out the molecular mechanism. Taken together, IL-1ra and IL-10 may mediate the inhibitory functions of AECM on EAC.

The above conclusions were also supported by other studies. For example, IL-1ra has been reported to suppress corneal transplant rejection and ocular inflammation (60–62) as well as to alleviate clinical symptoms in allergic eye disease (63). In addition, adenovirus-expressed IL-1ra relieves airway hyperresponsiveness symptoms and reduces allergic airway inflammation in asthmatic mice (64). A molecular mechanism study has indicated that IL-1ra, as a natural IL-1 receptor antagonist, downregulates the activity of IL-1 and APC function, thus preventing the activation and proliferation of antigen-stimulated CD4^+^ T cells, including Th2 cells, and inhibiting the subsequent allergic response (65, 66). The other anti-inflammatory factor, IL-10, is the main cytokine produced by Treg cells in allergic diseases. In asthmatic mice, adenovirus-expressed IL-10 inhibits airway inflammation (67). Furthermore, IL-10 has been reported to inhibit the proliferation of mitogen-induced T cells and the production of IL-1β and TNF-α pro-inflammatory cytokines (59).

It is worthy to point out that the effect of AECM on EAC mice is better than that of IL-ra or IL-10 or IL-1ra+IL-10, including the inhibitory effects on IgE release and MC activation and function, which needs further study. We think that other anti-inflammatory factors and immunomodulatory factors are also involved in the anti-allergic effects of AECM on EAC. In addition, the trophic factors, which are abundant in the AECM, may contribute to the repair of the damaged conjunctiva and thus provide additional treatment benefits.

Regarding the prospective applications of AECM, first, in terms of cell resource, hAEC are easily available, as they are derived from discarded term placenta, do not require invasive surgery, and have no ethical issues (68). In addition, hAEC have good proliferation ability and activity because the amniotic membrane originates from pluripotent epiblasts. Second, previous preclinical and clinical trials indicated that hAEC are safe; intravenous administration of hAEC does not result in hemolysis, allergic reactions, toxicity, or tumor formation (69). Third, such treatment using non-living cells has many advantages, such as ease of preparation, ease of preservation, low safety concerns, few ethical issues, and simple quality control process. AECM collection does not require pre-stimulation with TNF-α. More importantly, IL-1ra and IL-10 are effective and important paracrine factors in AECM, which provides a basis for quality control for future clinical applications of AECM. In addition, topical drug administration is a common and easy clinical administration route for the treatment of ocular disorders. Because AECM simultaneously targets multiple inflammatory signaling mediators with no side effect, it may represent a promising option for treating allergic diseases. Taken together, these unique properties suggest that AECM may represent a multitarget “drug” superior to a single immunosuppressive or anti-allergic drug. However, more clinical trials are required to assess and determine the safety and clinical benefits of AECM-based therapy on AC and other allergic diseases.

In summary, our findings demonstrated that AECM attenuates EAC symptoms and pathology in EAC mice. The anti-allergic effects of AECM are attributed to its inhibition of B-cell and MC activation and reduction of IgE and histamine release. At the molecular level, IL-1ra and IL-10 are potential crucial effectors in AECM for treating EAC. Such topical AECM therapy represents a novel strategy for treating AC and perhaps other allergic diseases.

## Data Availability Statement

The original contributions presented in the study are included in the article/**Supplementary Material**. Further inquiries can be directed to the corresponding authors.

## Ethics Statement

The collection and subsequent use of adult tissues were approved by the Human and Animal Research Ethics Committee of Renji Hospital, School of Medicine, Shanghai Jiaotong University. The animal study was reviewed and approved by the Committee on the Ethics of Animal Experiments of Tongji University (Permit Number: TJAA09620101).

## Author Contributions

BW, FG, HX, and G-TX designed the experiments and drafted the article. BW, FG, and LL performed the experiments and collected and analyzed data. JL collected human amnion. HX and G-TX critically reviewed the article and supervised the study. All authors contributed to the article and approved the submitted version.

## Funding

This work was supported by grants from the National Key R&D Program of China (2016YFA0101302, 2017YFA0104100, and 2020YFA0113100 to G-TX), Science and Technology Commission of Shanghai Municipality (18411953400 to G-TX), and Shanghai East Hospital Grant (ZJ2014-ZD-002 to G-TX).

## Conflict of Interest

The authors declare that the research was conducted in the absence of any commercial or financial relationships that could be construed as a potential conflict of interest.

## Publisher’s Note

All claims expressed in this article are solely those of the authors and do not necessarily represent those of their affiliated organizations, or those of the publisher, the editors and the reviewers. Any product that may be evaluated in this article, or claim that may be made by its manufacturer, is not guaranteed or endorsed by the publisher.
