# Supplementary material for: Conditioned Medium of Human Amniotic Epithelial Cells Alleviates Experimental Allergic Conjunctivitis Mainly by IL-1ra and IL-10
Source: Front Immunol. 2021 Nov 22;12:774601. doi: 10.3389/fimmu.2021.774601 (PMC8645696; doi:10.3389/fimmu.2021.774601)
Supplement: Supplementary file 1 [file DataSheet_1.docx]

Supplementary Materials

## Supplementary Figures


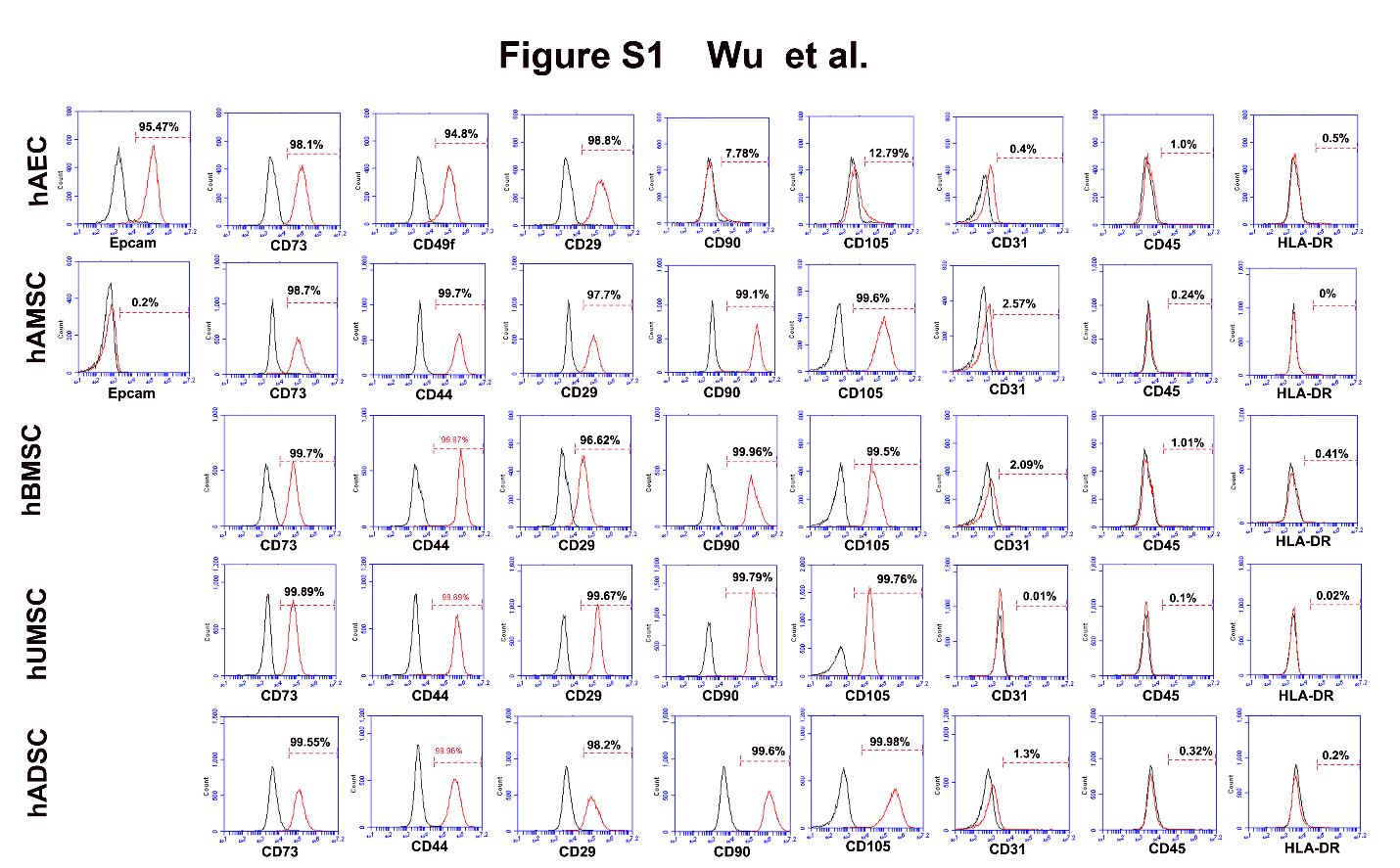


**Supplementary Figure 1.** Characterization of the tissue stem cells by flow cytometry with Epcam, CD49f, CD90, CD105, CD73, CD29, CD31, CD45 and HLA-DR.

**
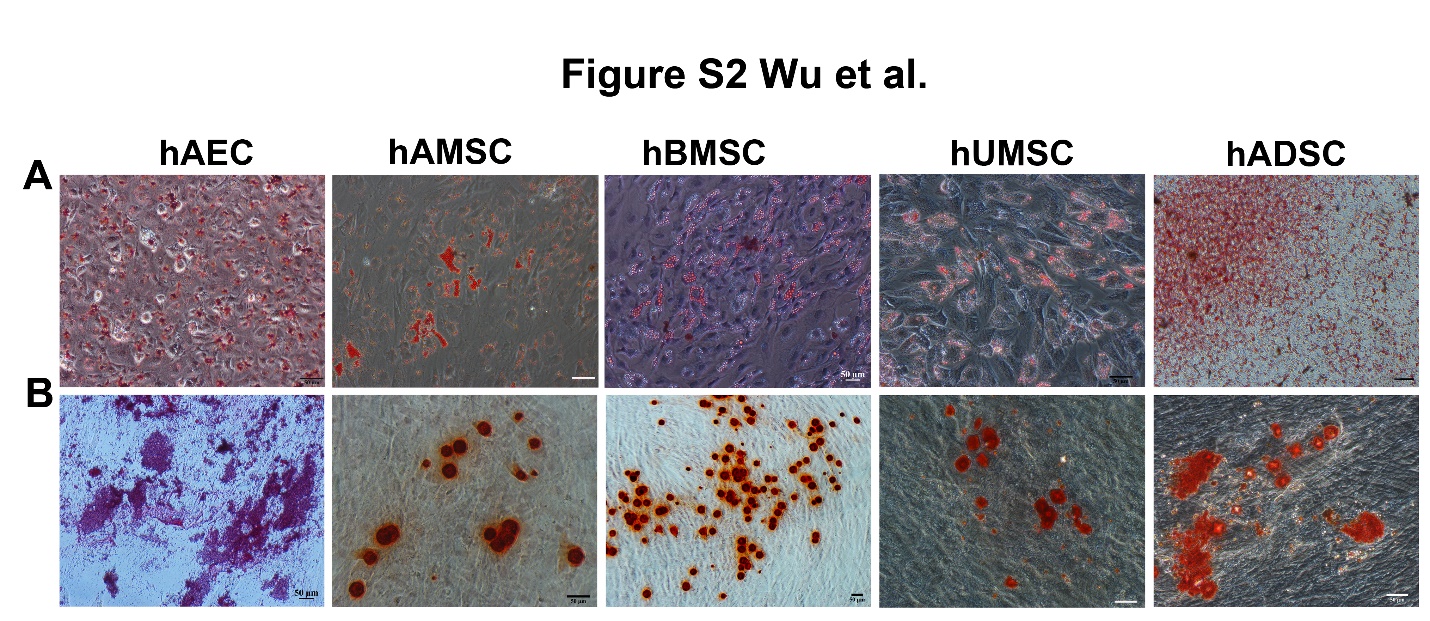
**

**Supplementary Figure 2.** *In vitro* osteogenic and adipogenic differentiation of tissue stem cells at P2

(**A**) Tissue stem cells were differentiated into adipocytes for 21 days and then analyzed by oil red-O staining. Scale bar:50μm.

(**B**)Tissue stem cells were differentiated into osteoblasts for 14 days and then analyzed by alizarin red staining. The top pattern was a control without alizarin red staining of differentiated cells. Scale bar: 50μm.


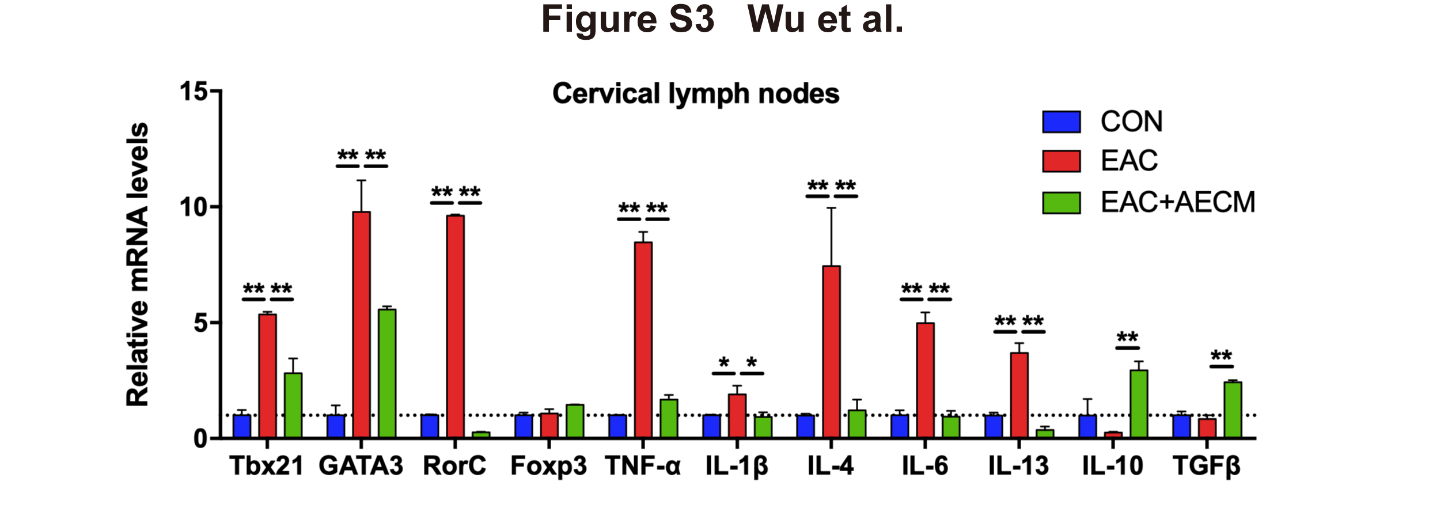


**Supplementary Figure 3.** Real-time PCR analysis of mRNA levels of cytokines and marker genes of immune cells in cervical lymph nodes of mice in each group. CON represents the normal control mice without SRW pollen treatment. The mRNA levels of genes in CON group were set as 1 and the data are expressed as the mean±SEM, n=3.


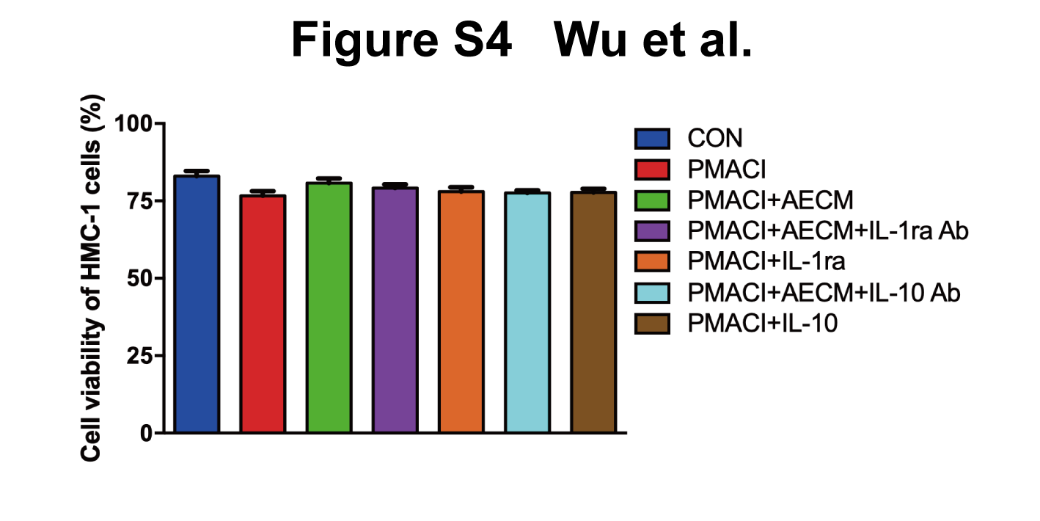


**Supplementary Figure 4.** Cell viability test. The cell viability of HMC-1 in different groups was assayed by trypan blue exclusion.

## Supplementary Tables

Supplementary Table 1. A table of normalized fluorescence signal intensity of 507 proteins in the AECM, AMSCM, BMSCM, UMSCM, and ADSCM.

Supplementary Table 2. A table of normalized fluorescence signal intensity of neurotrophic factors, growth factors, cell adhesion molecules (CAM) in the AECM, AMSCM, BMSCM, UMSCM, and ADSCM.

Supplementary Table 3. A table of normalized fluorescence signal intensity of anti-inflammatory factors, TGF β and other allergic-related factors in the AECM, AMSCM, BMSCM, UMSCM and ADSCM.

Supplementary Table 4. Primers used for real-time PCR analyses

Supplementary Table 5. The information of primary antibodies used in the study

## Supplementary Video

Supplementary Video 1. Typical scratching response of EAC mice after the challenge

## Supplementary Materials and Methods

**Isolation and culture of tissue stem cells**

hAEC were isolated from human amnion membrane of term placenta from women undergoing caesarean according to the protocol described in our previous study(1). In brief, the amniotic membrane was harvested and rinsed in HBSS solution, then the epithelium layer, the layer closest to the fetus, was stripped from the under layers of the amniotic membrane and was cut into small pieces 1 to 2 mm^2^. Then the tissues were oscillated and digested with 0.25% trypsin (Sigma-Aldrich, Darmstadt, Germany) in a water bath shaker for 30 min at 37℃, and then collagenase (0.1mg/ml, Sigma-Aldrich) for 1h at 37℃, and then DNase I (0.1mg/ml, Roche, Basel, Switzerland) for 10 min at room temperature (RT). Lastly, the cell pellet was filtered and the cells were resuspended in DMEM/F12 containing 10% FBS and 1% penicillin/streptomycin (P/S, all from Life Technology).

hAMSC was isolated from the under layers of the amniotic membrane removed by epithelial layer and digested and cultured in DMEM/F12 with 10% FBS like above hAEC isolation. hUMSC were isolated from human umbilical cord tissues obtained from women undergoing caesarean sections according to our previous method(2). In brief, the umbilical cord tissues were washed with PBS to remove blood, then the tissue were dissected to remove blood vessels, the remaining tissues were cut into small pieces 2-3mm^2^ and placed in culture dishes and added α-MEM medium containing with 10% FBS and 1% P/S into the dishes.14 days later, the cells would grow out from the tissues and digested by trypsin and cultured as hUMSC.

hBMSC were isolated from human bone marrow. First, bone marrow was collected from health volunteer after obtaining the informed consent and diluted with an equal volume of PBS. Then the bone marrow was isolated and centrifugated with a Ficoll Hypaque density gradient (MD Pacific Biotechnology) at 800g for 30 min, then aspirated the monocyte dense ring carefully into 15ml centrifuge tubes, and wash with PBS, and then resuspended and cultured with DMEM medium (low glucose) supplemented with 10% FBS. hADSC were isolated from adipose tissue obtained from health volunteer who had liposuction after obtaining the informed consent. First, adipose tissue was washed with HBSS solution to remove blood, then the adipose tissue was oscillated and digested with collagenase (0.1mg/ml) for 30 min at 37℃. Lastly, the cell pellet was filtered and the cells were resuspended in DMEM/F12 containing 10% FBS and 1% P/S.

**References**

1. Zhang J, Li H, Yang H, Lin J, Wang Y, Zhang Q, Gao WQ, Xu H. Human Amniotic Epithelial Cells Alleviate a Mouse Model of Parkinson's Disease Mainly by Neuroprotective, Anti-Oxidative and Anti-Inflammatory Factors. J Neuroimmune Pharmacol. 2020 Nov 9. eng. Epub 2020/11/10. doi:10.1007/s11481-020-09969-w. Cited in: Pubmed; PMID 33164162.

2. Yang M, Lin J, Tang J, Chen Z, Qian X, Gao WQ, Xu H. Decreased immunomodulatory and secretory capability of aging human umbilical cord mesenchymal stem cells in vitro. Biochem Biophys Res Commun. 2020 May 7;525(3):633-638. eng. Epub 2020/03/04. doi:10.1016/j.bbrc.2020.02.125. Cited in: Pubmed; PMID 32122651.
